# Surgical Wards, Service of Dr. E. Peace

**Published:** 1841-12-11

**Authors:** 


### CLINICAL REPORTS.

### PENNYSLVANIA HOSPITAL.

*Surgical Wards, Service of Dr. E. Peace.*

*Discharges.*—1. November 10, W. D. æt. forty-eight, admitted for a hydrocele cn the right side of five years standing. Tumour large, round, and diaphanous; the body of the right testicle, which was smaller than the left, having diminished in size by degrees as the hydrocele increased, was found, after considerable search, on the anterior superior surface of the sack. A reducible inguinal hernia, partially masked by the tumour, and previously unknown to the patient, was discovered in the right groin.

The hydrocele was tapped Oct. 3, about three pints of a turbid whitish fluid being withdrawn. On the third day thereafter, the part was attacked with a rather severe acute inflammation which subsided in three days under the influence of rest in bed, low, diet, and a lead water lotion. No fluid perceptible at the end of thirty-eight days.

In this case no resort was had to the ordinary means of exciting inflammatory action in the sack, nothing being done except the mere introduction of a trochar and canula, and the evacuation of the fluid. He appears, therefore to have been cured by the palliative operation.

2. Nov. 13. ———, a case of fungous tumour on the surface of the testicle not involving the proper structure of the organ, insensible, hard, rough, and irregular in surface; mottled in colour with dull red and brownish yellow patches, somewhat hemispherical in form, and, when first examined, about an inch in diameter, and rising half an inch above the integuments of the scrotum. The fungus had made its appearance nine days previous to his entrance. Treated for some time with emollient and fomenting cataplasms, without benefit, the tumour increased steadily during six weeks, until it became at least eighteen lines in diameter and fourteen in height. The part was then kept continually moist with lime water, and subjected to uniform compression—still without any marked improvement. Lastly, resort was had successively to astringents and escharotics; such as burnt alum,* sulphate of copper, and caustic potash. Under the action of these remedies, applied daily, every other day, or every two days, according to the amount of irritation produced,—compression being still sustained,—the tumour slowly disappeared. The caustic potash, which was the last article applied, appeared to act the most efficiently; since the slough was deeper, the absorption of the parts beyond it more rapid, and the pain of the application, although more severe, was more transient; and it created no irritation of the adjacent integuments; whereas, the sulphate of copper almost invariably inflamed the entire scrotum.

The pain excited by the dressings was felt only in the cord, neither the tumour nor the testicle appearing to suffer at any time during the course of the treatment. A small cavity was left in the spot occupied by the base of the tumour. This was stimulated to granulation by a strong solution of the sulphate of copper, and filled up in the course of ten days, when the man was discharged cured, the testicle being sound, although firmly adherent by its coats to the integuments in front. This case is deemed sufficiently rare and interesting to justify a more detailed report at a future period; and is therefore less fully narrated at the present time. E. H.

* An error of the press crept into the Clinical Report of last week, by which the reporter is made to accredit a friend with the introduction of burnt alum into the practice of the Pennsylvania Hospital. The phrase, of course, was intended to credit him only with its introduction in the treatment of otitis at that institution.

